# Supplementary material for: Splice-Junction-Based Mapping of Alternative Isoforms in the Human Proteome
Source: Cell Rep. Author manuscript; Available in PMC 2020 Jan 15. (PMC6961840; doi:10.1016/j.celrep.2019.11.026)
Supplement: 2 [file NIHMS1546469-supplement-2.zip › DF1/PXD010154/Testis/Testis_14_CCS_GSGQLRVQDVEVHLEDQMVLVHTTLPSQEVQALLEGTGR.pdf]

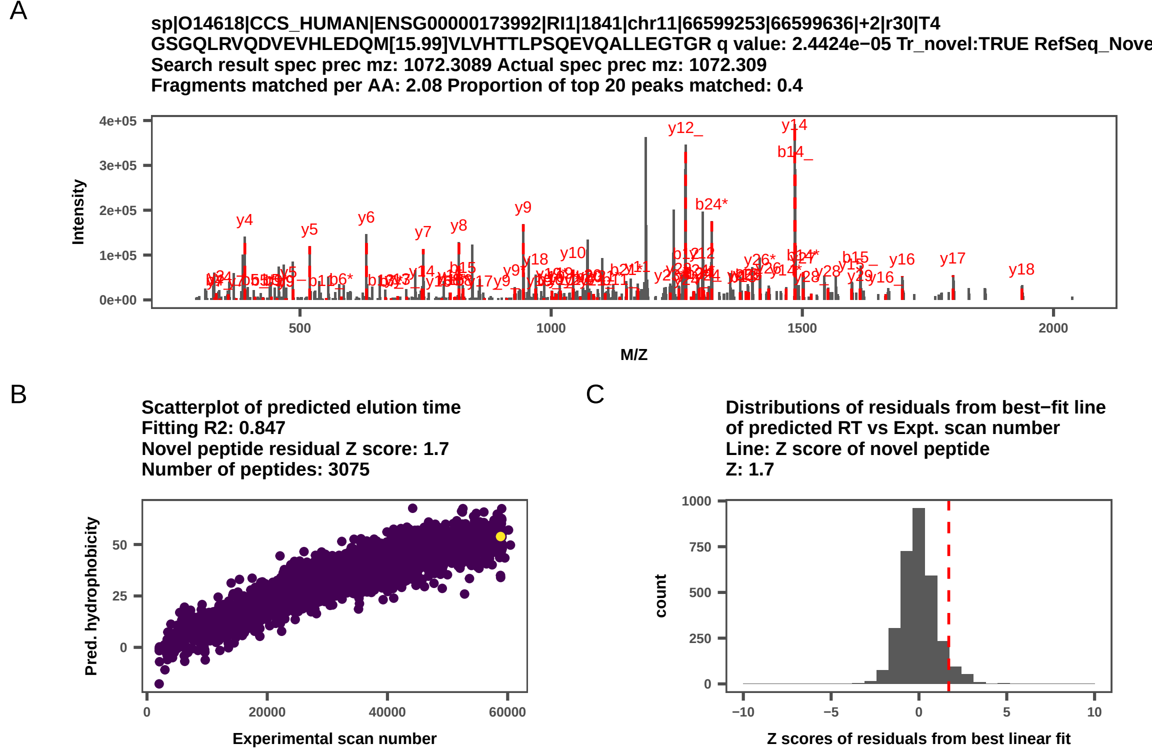
A
sp|O14618|CCS_HUMAN|ENSG00000173992|RI1|1841|chr11|66599253|66599636|+2|r30|T4
GSGQLRVQDVEVHLEDQM[15.99]VLVHTTLPSQEVQALLEGTGR q value: 2.4424e−05 Tr_novel:TRUE RefSeq_Nove
Search result spec prec mz: 1072.3089 Actual spec prec mz: 1072.309
Fragments matched per AA: 2.08 Proportion of top 20 peaks matched: 0.4
Intensity
4e+05
3e+05
2e+05
1e+05
0e+00
M/Z
500
1000
1500
2000
y4
y5
y6
y7
y8
y9
y10
y12_
y14
b14_
b24*
y16
y17
y18
B
Scatterplot of predicted elution time
Fitting R2: 0.847
Novel peptide residual Z score: 1.7
Number of peptides: 3075
Pred. hydrophobicity
50
25
0
Experimental scan number
0
20000
40000
60000
C
Distributions of residuals from best−fit line
of predicted RT vs Expt. scan number
Line: Z score of novel peptide
Z: 1.7
count
1000
750
500
250
0
Z scores of residuals from best linear fit
−10
−5
0
5
10
